# An Integrated System of Braden Scale and Random Forest Using Real-Time Diagnoses to Predict When Hospital-Acquired Pressure Injuries (Bedsores) Occur

**DOI:** 10.3390/ijerph20064911

**Published:** 2023-03-10

**Authors:** Odai Y. Dweekat, Sarah S. Lam, Lindsay McGrath

**Affiliations:** 1 Department of Systems Science and Industrial Engineering, Binghamton University, Binghamton, NY 13902, USA; 2 Wound Ostomy Continence Nursing, ChristianaCare Health System, Newark, DE 19718, USA

**Keywords:** hospital-acquired pressure injuries, bedsores, pressure injuries, pressure ulcer, Braden Scale, machine learning, integrated systems, predictive analytics

## Abstract

Background and Objectives: Bedsores/Pressure Injuries (PIs) are the second most common diagnosis in healthcare system billing records in the United States and account for 60,000 deaths annually. Hospital-Acquired Pressure Injuries (HAPIs) are one classification of PIs and indicate injuries that occurred while the patient was cared for within the hospital. Until now, all studies have predicted who will develop HAPI using classic machine algorithms, which provides incomplete information for the clinical team. Knowing who will develop HAPI does not help differentiate at which point those predicted patients will develop HAPIs; no studies have investigated when HAPI develops for predicted at-risk patients. This research aims to develop a hybrid system of Random Forest (RF) and Braden Scale to predict HAPI time by considering the changes in patients’ diagnoses from admission until HAPI occurrence. Methods: Real-time diagnoses and risk factors were collected daily for 485 patients from admission until HAPI occurrence, which resulted in 4619 records. Then for each record, HAPI time was calculated from the day of diagnosis until HAPI occurrence. Recursive Feature Elimination (RFE) selected the best factors among the 60 factors. The dataset was separated into 80% training (10-fold cross-validation) and 20% testing. Grid Search (GS) with RF (GS-RF) was adopted to predict HAPI time using collected risk factors, including Braden Scale. Then, the proposed model was compared with the seven most common algorithms used to predict HAPI; each was replicated for 50 different experiments. Results: GS-RF achieved the best Area Under the Curve (AUC) (91.20 ± 0.26) and Geometric Mean (G-mean) (91.17 ± 0.26) compared to the seven algorithms. RFE selected 43 factors. The most dominant interactable risk factors in predicting HAPI time were visiting ICU during hospitalization, Braden subscales, BMI, Stimuli Anesthesia, patient refusal to change position, and another lab diagnosis. Conclusion: Identifying when the patient is likely to develop HAPI can target early intervention when it is needed most and reduces unnecessary burden on patients and care teams when patients are at lower risk, which further individualizes the plan of care.

## 1. Introduction

Pressure injuries (PIs) are the second most common diagnosis in healthcare system billing records in the United States and account for 60,000 deaths annually. The annual cost of PIs is more than $26.8 billion, which accounts for 25% of wasteful spending related to failures in healthcare delivery. The cost to an individual facility for a stage 3, 4, or unstageable injury is between $75,000 and 150,000 per injury, with an average malpractice claim of $250,000. Hospitals in the US are penalized a total of 1% of total Centers for Medicare and Medicaid Services (CMS) reimbursements if their hospital-acquired condition rates fall below the 25th percentile among all the hospitals [1].

Hospital-Acquired Pressure Injuries (HAPIs) are one classification of PIs and indicate injuries that occurred while the patient was cared for within the hospital. In acute care settings, the average is 2.1 per 1000 patients, with 10–20% occurring in critical care areas; skilled nursing facilities have an average incidence of 20–30% [1].

**Figure 1:**
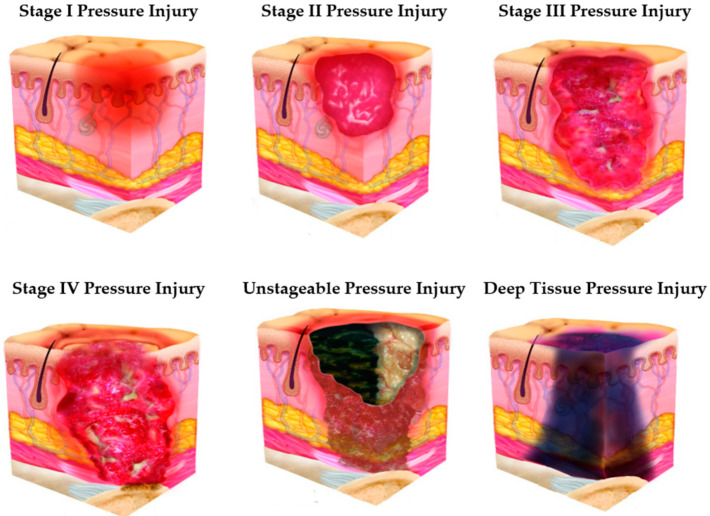
PI stages/categories (adapted from [2]).

PIs can happen anywhere on the body but are typically observed over bony prominences or under medical equipment. PIs are further categorized by the level of tissue exposed using a staging system, as follows: Stage 1: PIs present as intact skin with a localized area of non-blanchable erythema. Stage 2: partial-thickness skin loss with exposed dermis or may be a serum-filled blister. Stage 3: full-thickness skin loss in which adipose tissue is visible. Stage 4: “full-thickness skin and tissue loss” [2], which exposes underlying structures such as fascia, muscle, tendon, or bone. Unstageable injuries are injuries where the extent of skin and tissue loss is obscured by slough or eschar. In contrast, a Deep Tissue Pressure Injury (DTPI) is a localized area of non-blanchable deep red, maroon, or purple discoloration which may evolve rapidly as the extent of the injury is revealed [2], as presented in Figure 1.

Most PIs are preventable and can be prevented by identifying risk early and implementing prevention strategies. The first step in PI prevention is use of a standardized risk assessment tool to identify at-risk patients and their risk factors [2]. The currently available validated risk assessment tools, such as the Braden Scale [3,4,5], have a high sensitivity, but also a high False Positive Rate (FPR). The high FPR is difficult to manage for caregivers due to the level of individualized care that is provided to the patients. Specifically, in ICU or step-down settings where patients are at risk for a prolonged period, the individualized care can be burdensome for the patient and cause disruptions in comfort and sleep.

Machine Learning (ML) has been utilized to predict HAPI patients before occurrence using patients’ Electronic Health Records (EHR) as an adjunct to clinical assessment, which can further narrow down which patients are at risk [6,7,8,9]. In the last 15 years, 30 studies have answered who will develop HAPIs by utilizing classic machine and deep learning algorithms [10,11,12,13,14,15,16,17,18,19,20,21,22,23,24,25,26,27,28,29,30,31,32,33,34,35,36,37,38,39]. Two studies adopted Grid Search (GS) to optimize the hyperparameters of ML [10,22]. In most studies, random oversampling, undersampling, and Synthetic Minority Oversampling Techniques (SMOTE) were adopted as oversampling techniques [10,11,12,13,14,15,16,17,18,19,20,21,22,23,24,25,26,27,28,29,30,31,32,33,34,35,36,38]. Nevertheless, one study adopted cost-sensitive learning [22] to deal with the unbalanced dataset. The most used algorithms were Random Forest (RF) [10,12,13,14,15,16,19,21,22,25,30,31,32,36,38,39], Logistic Regression (LR) [10,11,14,16,18,20,21,22,23,24,25,26,27,29,30,31,32,34,36], Decision Tree (DT) [15,16,21,23,25,26,27,28,31,32,36], Support Vector Machine (SVM) [10,12,21,22,25,26,27,28,30,38,39], Multilayer Perception (MLP) [12,13,15,21,22,25,28,30,38], k-Nearest Neighbor (KNN) [15,30,32], and Linear Discriminant Analysis (LDA) [15].

Other studies were conducted after the occurrence of HAPI to predict the HAPI stages using Convolutional Neural Networks (CNN) [40,41,42,43,44,45]. PI images were used as input to develop a classification model. As illustrated in Figure 1, six stages of PIs are classified by Fergus et al., Yilmaz, A et al., and Yilmaz, B et al. [41,43,44]. Similarly, Ay et al. classified the first four stages [40]. Liu et al. classified the first two stages [42]. On the other hand, Matsumoto et al. classified four different patterns within DTPI [45].

Predicting HAPI stages does not indicate HAPI’s occurrence. The initial stage of HAPI may occur within a few days or weeks following admission. Consequently, prediction of the stage of HAPI does not aid nurses in differentiating the severity and urgency of the expected stages. Therefore, there is a need for a method to anticipate when HAPIs will occur in patients who are prone to develop HAPIs at a given time to deliver early interventions to patients who are likely to develop PIs at a specific point in time (i.e., highest risk cases). Alternatively, predicting who will develop HAPIs provides incomplete information for the clinical team because knowing who will develop HAPIs does not help differentiate the severity among those predicted patients.

Until now, studies have predicted who will develop HAPIs or HAPI stages. However, no studies have investigated when HAPIs happen for predicted at-risk patients. This research aims to develop a hybrid RF and Braden Scale integrated system to predict when the patient will likely develop HAPI to provide early intervention for the highest-risk patients. The suggested model considers the changes in patients’ diagnoses from admission until HAPI occurrence.

This research is structured as follows: Section 2 summarizes the contribution of this research. Section 3 describes the research methodology. Section 4 summarizes the results. Section 5 discusses the suggested approach’s outputs, implications, and limitations. Lastly, Section 6 provides the conclusions and future work.

## 2. Contributions

**What is already known?**

- Research studies have predicted who will develop HAPIs or predicted HAPI stages. However, no studies have investigated when HAPIs happen for predicted at-risk patients by considering the changes in the status of patients from admission until HAPI occurrence.
- Predicting who will develop HAPIs provides incomplete information for the clinical team because knowing who will develop HAPIs does not help differentiate the severity of those predicted patients.

**What does this paper contribute to the wider global clinical community?**

- This research aims to develop a hybrid random forest, grid search, and Braden Scale integrated system to predict when the patient will develop HAPI to provide early intervention for the highest-risk patients.
- This research is the first study that predicts when HAPIs occur by considering the changes in the status of patients from admission until HAPI occurrence.
- This research prioritizes who is likely to develop HAPIs at a specific point in time (high risk) and it helps with better staff planning to provide intervention for high-risk patients.
- This research measures the financial impact of the proposed model.

## 3. Research Methodology

### 3.1. Study Design and Setting

The scope of this study includes patients 18 years old or older who developed HAPIs during their inpatient care episode at ChristianaCare hospital located in Delaware, USA. The time frame was identified through the discharge date between May 2020 and February 2022. Labor and delivery, emergency, and psychiatric visits are out of scope.

### 3.2. Data Source

**Figure 2:**
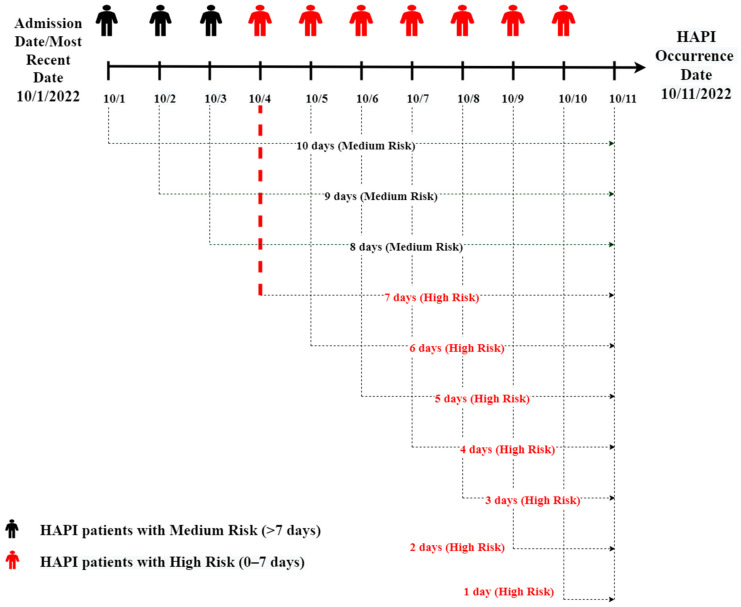
Days of HAPI data collection from admission until HAPI occurrence.

HAPI patients (N = 485) and timing of HAPI occurrence were identified from Wound, Ostomy, Continence (WOC) nurse documentation that validates pressure injuries as HAPIs. Multiple factors for HAPI patients were extracted daily from their EHR starting from admission until HAPI occurrence (N = 4619 records; each record represents a day in patient stay) to help predict timing of HAPI. The variables included demographics, diagnosis, labs, vitals, medical devices, and nursing assessment results that included the Braden Risk Assessment. Data were extracted daily to monitor changing patient values to predict the number of days until HAPI occurrence. Figure 2 explains how data were extracted from admission until HAPI occurs; the days needed for HAPI to develop are shown below.

### 3.3. Variables

The goal of this study is to predict when patients are expected to develop HAPIs using multiple variables. Sixty variables that include the Braden Risk Assessment subscales were used as inputs for a model to predict HAPI timing. The HAPI timing was identified as the number of days to develop HAPIs [8,9,10,11,12,13,14,15,16,17,18,19,20,21,22,23,24,25,26,27,28,29,30,31,32,33,34,35,36,37,38,39,46]. These variables are shown in Table 1 and selected through literature survey and clinician’s feedback [8,9,10,11,12,13,14,15,16,17,18,19,20,21,22,23,24,25,26,27,28,29,30,31,32,33,34,35,36,37,38,39,46]. The values of these variables were extracted daily because some of them could change every shift or every day. The days needed to develop HAPIs are highest on admission and drop to 0 when HAPI develops.

The days to develop HAPIs (model target) were divided into two categories: high risk, i.e., within the next 7 days, and medium risk, i.e., more than 7 days. The high-risk population would benefit from focused prevention actions and intervention to reduce their likelihood of developing HAPI. The medical teams confirmed this categorization of days, where 7 days is an adequate period to provide earlier preventive actions; high-risk patients receive immediate intervention attention, and medium-risk patients receive later interventions. The distribution of high-risk and medium-risk patients is summarized in Table 2.

### 3.4. Model Development

The dataset was split into 80% for training with 10-fold cross-validation and 20% for testing. RF was used as the prediction algorithm for the model. The RF algorithm that was proposed in 2001 [47] is a combination of many randomized decision trees in which their predictions are averaged or combined using a voting approach. The RF algorithm has demonstrated excellent performance in the literature when the number of variables is high compared to number of observations [48]. Furthermore, RF models provide high-level explanations of variable importance to illustrate how variables contributed to the prediction of the class or target variable. One of the disadvantages of RF models is that it is difficult to interpret individual predictions and can be perceived as a black box. The pseudocode for the RF algorithm is provided in Algorithm 1.
**Algorithm 1.** Pseudocode for Random Forest Algorithm [49].To generate c classifiers： **for**
i=1 to c **do**  Randomly sample the training data D with replacement to produce Di  Create a root node, $N_{i}$ that contains $D_{i}$  Call Build Tree ($N_{i}$) **end for** **Build Tree(N):** **if**
N contains instances of only one class **then**  **return** **else**  Randomly select x% of the possible splitting features in N  Select the variable F with the highest information gain to split on  Create f child nodes of $N,\;N_{1},\;\ldots ,\;N_{f}$, where F has f possible values $\left(F_{1},\;\ldots ,\;F_{f}\right)$  **for**
i=1 to f **do**    Set the contents of $N_{i}$ to $D_{i}$, where $D_{i}$ is all instances in N that match $F_{i}$    Call Build Tree ($N_{i}$)  **end for** **end if**

The Braden Risk Assessment (i.e., Braden Scale) is a skin assessment to predict patients’ risk of developing pressure injuries [3,4,5]. It considers six main factors: sensory perception, moisture, activity, mobility, nutrition, friction, and shear. Each one of these factors can take a value between 1 and 4, except for friction and shear, which takes values between 1 and 3; 1 is the worst condition, and 4 is the best condition. The sum of these categories’ scores is called the Braden Score. When the score is higher than 18, the patient is considered at low risk for developing a pressure injury. When the score is less than 18, the patient is considered high risk. The six subscales of the Braden Scale were used as input variables for the RF prediction model.

**Figure 3:**
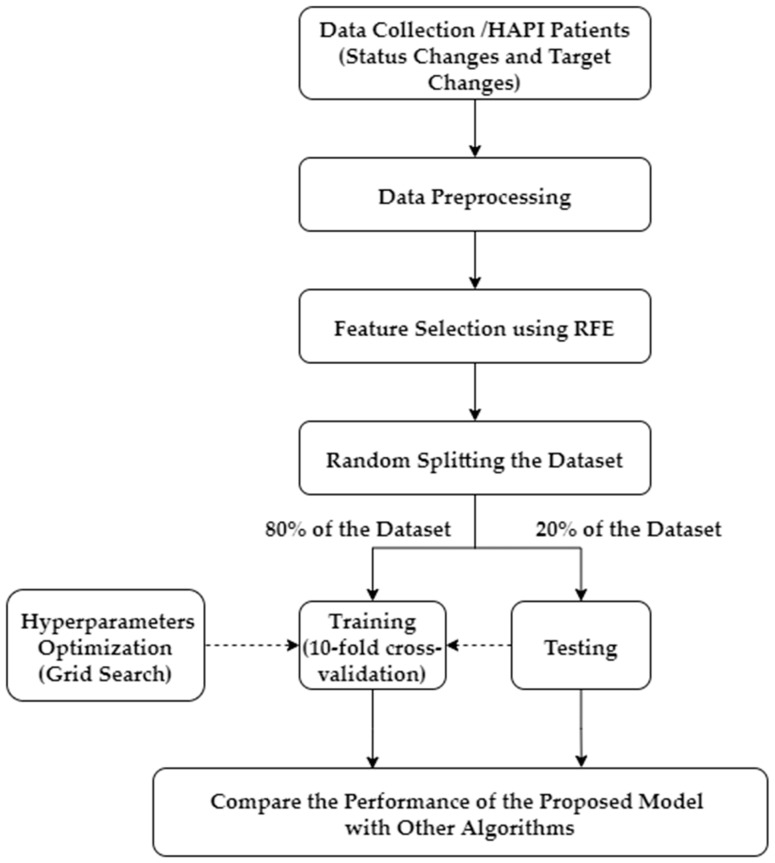
Research framework of the proposed approach.

Figure 3 shows the research framework proposed in this study, which starts with data collection for HAPI patients, followed by data preprocessing that includes data cleaning and handling missing values. Mean imputation was used to impute missing data. RFE was used to select top important variables for the prediction model.

The dataset was split into 80% for training and 20% for testing. Ten-fold cross-validation was used in training the RF model to avoid overfitting. Furthermore, the RF parameters were optimized using GS for best classification performance [50]. Finally, the performance of the model was compared to the testing set. The model training and testing was repeated for 50 iterations; at every iteration, the training/testing data split was performed randomly. The RF model performance was compared with seven commonly applied algorithms in HAPI research that include: SVM, MLP, KNN, LDA, DT, LR, and Adaptive Boosting (AdaBoost) [10,11,12,13,14,15,16,17,18,19,20,21,22,23,24,25,26,27,28,29,30,31,32,33,34,35,36,37,38,39].

Performance metrics used for evaluation were sensitivity, Geometric Mean (G-mean), Area Under the Curve (AUC), and FPR. Analysis of Variance (ANOVA) was performed to conduct statistical comparison between models. Confidence levels were calculated at a 0.05 error margin for the 50 iterations. Additionally, the dataset was not highly imbalanced; therefore, no balancing techniques were applied.

In GS, the algorithm searches exhaustively through a predefined manual subset of the hyperparameter space of RF [9]. The hyperparameters used for the GS are: maximum depth [none, 5, 8, 15, 50]; minimum samples leaf [1, 2, 4]; minimum samples split [2, 5, 10]; bootstrap [true, false]; the number of trees/estimators [200, 400, 500, 800, 1000]; maximum number of features RF allowed in the individual tree is [‘auto’, ‘sqrt’]; and random state is fixed at 7. The total number of combinations for the experiments is 900. GS is performed with 10-fold cross-validation. The GS resulted in the selection of the following optimal parameter values: maximum depth is 15; bootstrap is false; maximum number of features is sqrt; min samples leaf is 1; min samples split is 2; and number of trees is 400. The proposed model was developed using Python 3.9.0.

### 3.5. Performance Metrics

Performance metrics used in this study included sensitivity, G-mean, AUC, and FPR. Refer to Table 3 for detailed explanation of the confusion matrix metrics that include False Negative (FN), False Positive (FP), True Negative (TN), and True Positive (TP). FN represents high-risk records predicted as medium risk. FP represents medium-risk records predicted as high risk. TN represents medium-risk records predicted as medium risk. TP represents high-risk records predicted as high risk.

Sensitivity is the ratio of TP to all actual high-risk records, as shown in Equation (1). FPR measures the probability of medium-risk records predicted as high risk, as shown in Equation (2). The G-mean measures the balance between classification performances on both the medium-risk and high-risk records, as shown in Equation (3). Lastly, AUC measures the ability of the model to classify medium-risk and high-risk records.

(1) $$\mathrm{Sensitivity}=\frac{\mathrm{TP}}{\mathrm{TP}+\mathrm{FN}}\;\times \;100\%$$

(2) $$\mathrm{FPR}=\frac{\mathrm{FP}}{\mathrm{FP}+\mathrm{TN}}\;\times \;100\%$$

(3) $$G-\mathrm{mean}=\sqrt{\left(\frac{\mathrm{TP}}{\mathrm{TP}+\mathrm{FN}}\right)\times \left(\frac{\mathrm{TN}}{\mathrm{TN}+\mathrm{FP}}\right)}\times \;100\%$$

## 4. Results

The significant advantage of this research is the development of an integrated system of RF and Braden Scale, which predicts HAPI time by considering the changes in patients’ diagnoses from admission until HAPI occurrence. RFE is used to select the 43 most dominant factors among the 60 factors, as shown in bold in Table 1. The dataset is separated into 80% training vs. 20% testing. The hyperparameters of RF are optimized using GS, then the proposed model (GS-RF) is validated with the seven most common algorithms. RF measures the importance of risk factors that affect the HAPI time frame. Statistical analysis is conducted to demonstrate the statistical significance of the proposed approach.

GS-RF achieved the best sensitivity (89.33 ± 0.49), AUC (91.20 ± 0.26), G-mean (91.17 ± 0.26), and FPR (6.94 ± 0.31) compared to the most common classification algorithms used to classify HAPI time (MLP, AdaBoost, KNN, DT, LR, SVM, and LDA), as presented in Table 4. Ten-fold cross-validation was conducted to avoid overfitting. Moreover, 50 experiments were conducted for each with a 95% Confidence Level (CL). It is worth mentioning that no oversampling method was adopted because the percentage of patients who developed HAPI within 7 days was 36.76% vs. 63.24% of patients who developed HAPI after more than 7 days.

**Figure 4:**
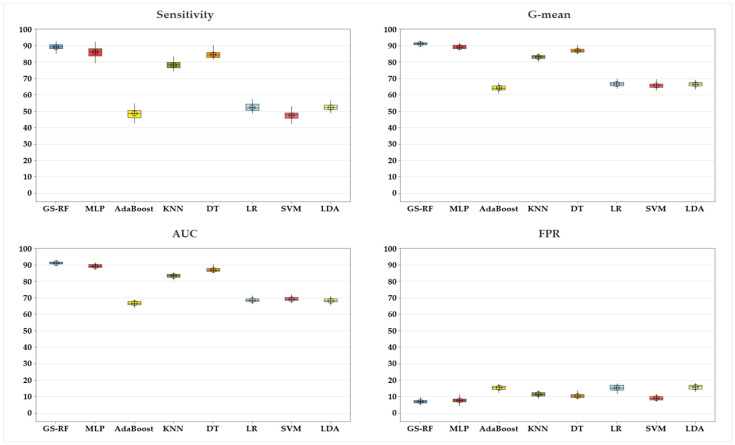
Results for GS-RF vs. other algorithms.

ANOVA was conducted to assess if there is a statistical difference between all algorithms by testing for differences in means, as shown in Table 5. The *p*-values for sensitivity, AUC, G-mean, and FPR are all less than 0.05, which indicate a statistically significant difference between the techniques. Figure 4 depicts the mean and confidence interval for GS-RF in comparison to other methods for sensitivity, AUC, G-mean, and FPR. It is observed that GS-RF is the most effective approach.

**Figure 5:**
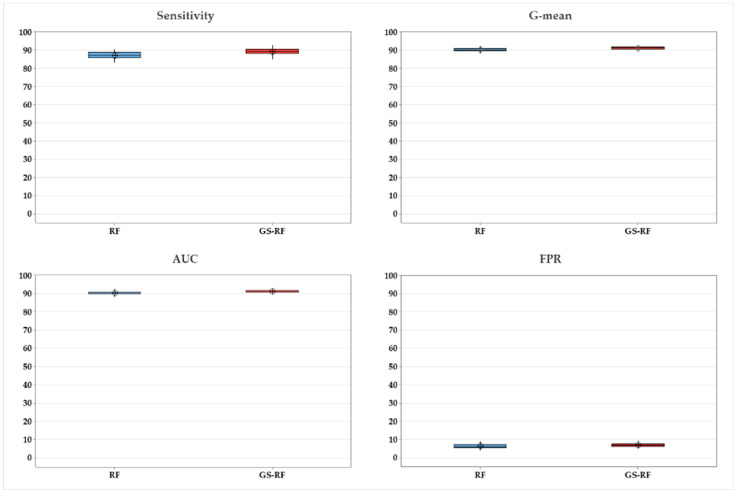
Comparison between GS-RF and RF.

The *t*-test was utilized to demonstrate that there is a statistical difference between the RF and the optimized RF (GS-RF) in terms of sensitivity, AUC, and G-mean (*p*-value is less than 0.05), as demonstrated in Table 5 and illustrated in Figure 5.

**Figure 6:**
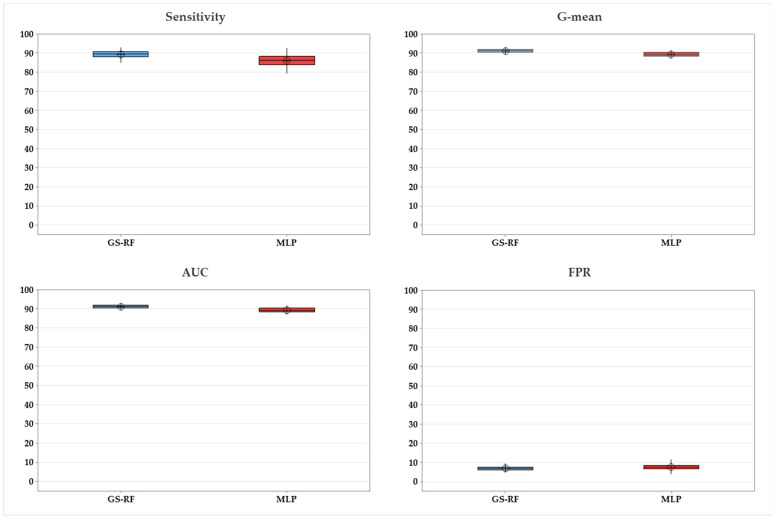
Comparison between GS-RF and MLP.

It is seen from Figure 4 that MLP has decent performance metrics in comparison to the other algorithms. The *t*-test is therefore conducted between GS-RF and MLP. Table 5 demonstrates statistical significance (*p*-value is 0.05) between GS-RF and MLP in terms of sensitivity, AUC, G-mean, and FPR, as shown in Figure 6.

**Figure 7:**
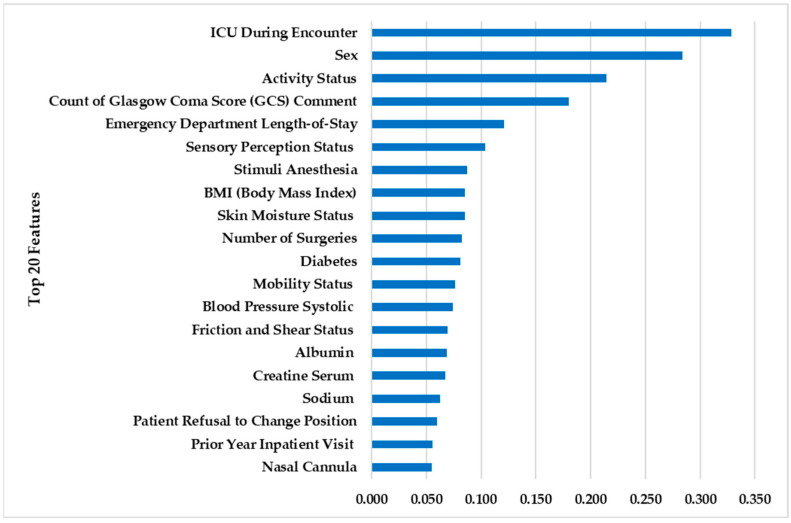
Feature importance for the top 20 risk factors that affect HAPI time.

RFE selects the 43 most influential factors to develop the model, which are highlighted in bold in Table 1. To examine the impact of each risk factor in predicting HAPI time, the significance of each factor was calculated using Gini impurity in RF. The significance of the top 20 risk variables in predicting HAPI time is depicted in Figure 7. Examples include ICU during encounter, Stimuli Anesthesia, and five Braden subscales.

## 5. Discussion

This research develops an integrated system of machine learning and Braden Scale to predict HAPI time by considering the changes in patients’ diagnoses from admission until HAPI occurrence. The developed method achieved the best AUC (91.20 ± 0.26) and G-mean (91.17 ± 0.26) compared to the seven algorithms. The proposed model predicts the time HAPI will develop using a dynamic approach. The addition of ML to Braden Scale can significantly review more of the risk factors while continuing to use objective and subjective data from Braden risk assessment tool performed by bedside clinicians, which results in a complete clinical picture and evaluation of risk.

The standard bedside risk assessments can provide the patient level of risk in relation to current and specific risk factors; however, they are unable to account for historical factors or changes in data over time [8,46] due mainly to limited resources at time of assessment. In areas of the healthcare system where length-of-stay can be prolonged, which provides an individualized HAPI prevention plan of care which can be burdensome to the patient (sleep disturbances, discomfort, cost) and cause burnout amongst caregivers [2]. The addition of timing of the HAPI is important because it can lead to escalation and de-escalation of a care plan in response to current level of risk, changing conditions over time, and historical factors able to be assessed by the addition of ML algorithms [9]. Understanding not only who is at risk but further narrowing down when they are most at risk will allow for prioritization of care, reduced cost, consideration in staffing models, and increased patient comfort [9]. Staff can prioritize patient care; for instance, if one nurse has five patients and four of them are at risk based on standard bedside risk assessment but only if one is at risk based on the addition of time, that nurse can prioritize care knowing upon whom the interventions should be targeted [9].

Healthcare systems are currently in a staffing crisis and nurse-to-patient ratios (N:P) are higher than in the past (i.e., nurses serve a higher number of patients than in the past). The addition of the time frame when injury is most likely to happen reduces the volume of patients who require additional interventions, and those patients who require additional interventions for HAPI prevention can be included when assigning nursing and support staff, which matches an individual patient with the right staffing model.

**Figure 8:**
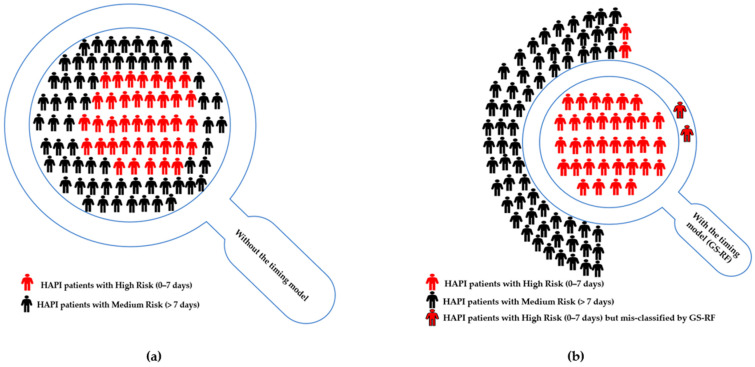
Illustration of a sample of 100 patients predicted to have HAPI, where 38 of them are at high risk (0–7 days). (**a**) **Without the timing model**: conducting prevention actions for all 100 patients (i.e., detection prevalence) predicts 38 patients with high risk (0–7 days) (i.e., sensitivity) correctly; (**b**) **GS-RF (with Braden)**: conducting prevention actions for 36 patients (i.e., detection prevalence) predicts 34 patients with high risk (0–7 days) (i.e., sensitivity) correctly.

A comparison of the current use of validated pressure injury risk (i.e., without the timing model) and the integrated model can be conducted to measure the financial impacts on a group of 100 patients identified as HAPI, which assumes 38 patients are at high risk (0–7 days) based on the percentage of at-risk patients in Table 2. Without the timing model, all 100 patients are considered at high risk for HAPI development, which means prevention actions are provided for all 100 patients. The addition of RF to Braden Scale reduces the number to 35–37 patients, which is known as detection prevalence. Detection prevalence indicates the percentage of patients identified as high risk (0–7 days) by the model. This determines the number of nurses needed to provide preventive actions for the suspected high-risk HAPI cases (i.e., TP and FP) [46,51]. Therefore, the savings is between 63 and 65 patients who require preventive actions per day. The National Pressure Injury Advisory Panel (NPIAP) reports that the average cost of prevention is $50–100 daily per patient [1] in terms of time, labor, products, pressure-reducing surfaces, and devices, which is significantly less than the cost of an injury but is not reimbursed by CMS. As a result, the developed model saves between $1,149,750 and 1,186,250 per year when the daily cost is $50 per patient, whereas it saves between $2,299,500 and 2,372,500 per year when the daily cost is $100 per patient, as summarized in Table 6 and graphically presented in Figure 8.

From a bedside clinician’s perspective, this reduction in volume of those identified as being at risk allows resources to be targeted where they will have the most benefit and can reduce the overall cost of care. From a WOC nurse or unit leadership perspective, prevention is top of mind, and the reduction in the total number of patients at risk for pressure injuries allows for monitoring of care plans, increased support to staff, and targeted education for patient-specific risk factors. Additionally, the patient will benefit through a reduction of unnecessary and often burdensome prevention interventions. An example of clinical application is in the sleep protocol, in which clustering care allows for longer periods of rest overnight; currently, patients at risk for pressure injuries are not eligible for sleep protocol due to turns being required every 2 hours. A reduction in the number of patients identified as being at risk could benefit a larger number of patients by allowing for longer periods of uninterrupted rest.

Through targeting pressure injury prevention strategies to those who are most at risk, the results will likely show a reduction in pressure injuries at the systemwide level. This reduction can go on to have a positive impact on length-of-stay and 30-day readmission rates. According to the NPIAP, patients who develop a HAPI have a longer length-of-stay by 4.31 days and a 30-day readmission rate of 22.6% compared to 17.6% for those without HAPI [2]. Therefore, HAPI prevention in patients could reduce length-of-stay.

The addition of time will allow for prioritizing which patients will receive the most benefit out of prevention interventions and when it will be most appropriate. For many years, best practice is to turn every 2 hours, but that can be uncomfortable for patients, especially at night; however, recent studies show that there is little difference in healthy subjects between every 2 hours, 3 hours, or 4 hours turning on pressure redistributing surfaces [52,53,54]. The question has always been how to match the individual patient with the right intervention. Considering the time frame for likely HAPI development can lead to customization of turning schedules and reduce the burden on patient and staff when the intervention may not be needed as frequently at certain points during the stay.

The use of real-time data along with changes in the patient’s condition allows for a more accurate and holistic view of the patient, which leads to an increased understanding of patient risk. Current risk assessment tools take static snapshots of patient risk [3,4,5] and consider only what the patient is doing currently or at one point in time. Bedside risk assessments use this model because the staff who cares for the patient changes and can only provide information on their assessment. The current ML model looks at diagnosis at admission without considering changes in patient condition over time [10,11,12,13,14,15,16,17,18,19,20,21,22,23,24,25,26,27,28,29,30,31,32,33,34,35,36,37,38,39], such as current bedside risk assessments.

The factors considered are pulled directly from EHR and consider current assessment. ICU during encounter, emergency department length-of-stay, number of surgeries, as well as prior year inpatient visits shed light on the overall patient health status. Certain areas of the current nursing bedside assessment are also pulled into the risk model, which include activity status, Glasgow coma score/sensory perception, moisture, mobility, friction and shear, as well as lab values to indicate nutritional status, as presented in Figure 7. This combination of acute assessment by the bedside team and the dynamic model provides a partnership between the direct care provider and ML approach.

**Figure 9:**
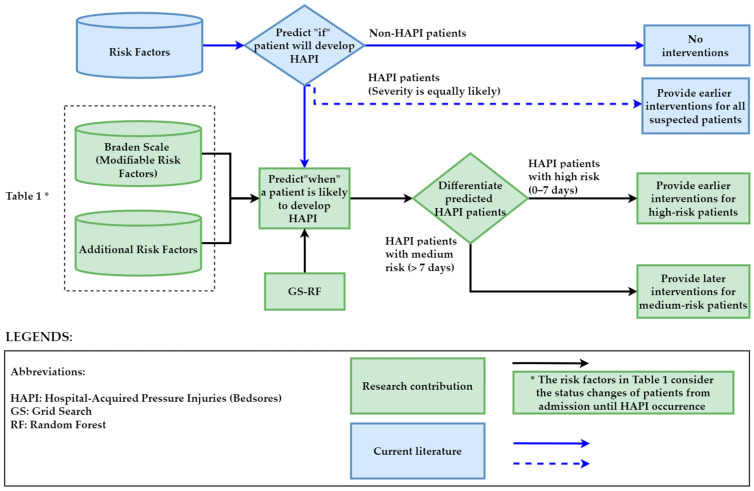
Research contribution and model implementation.

Current use of validated pressure injury risk tools performed by the bedside nurse or care team results in a large population of patients identified as being at risk to develop pressure injuries. Nursing and other bedside caregivers are then required to triage needs and allocate resources based on level of risk and nursing judgement. Despite using validated tools and ever-increasing clinical and technological advances in the prevention of pressure injuries, HAPI rates have continued to climb nationwide according to the United States Agency for Healthcare Research and Quality (AHRQ) [55]. Therefore, the risk factors of the patients suspected of developing HAPI by validated pressure injury risk tools can be fed to the developed method. As a result, it provides an indication as to not only who will develop HAPIs but also prioritizes who is likely to develop HAPIs at a specific point in time and provides targeted prevention for high-risk patients (0–7 days), as shown in Figure 9.

The model will allow for review of current and historical data; however, this limits the inter-rater reliability of current risk assessment scores and subjective assessments. Currently the model pulls from specific fields in the EHR; however, assessment and inclusion of expert assessment may benefit the model in terms of increased accuracy of the data. Examples include use of mobility assessment from the physical therapist and consultation or nutritional data from the registered dietitian. The current disadvantage of the developed model from a practical point of view is that some patients may be at risk for their entire stay based on factors that cannot be modified or changed, and many critical care patients may be hospitalized for a prolonged period of time. Lastly, the suggested approach is constructed using patients’ EHR from ChristianaCare hospital; therefore, it may need retraining if applied to another population at a different healthcare facility.

## 6. Conclusions and Future Work

HAPI is the second most common diagnosis in the US healthcare billing records and causes 60,000 fatalities annually [1]. It can lead to fines, lawsuits, and a loss of trust in the healthcare system [1,46]. Patient safety depends on HAPI prevention. Researchers have used ML to predict HAPI development or phases. This is insufficient and incomplete information for the clinical team because knowing who will develop HAPIs does not help distinguish severity. Preventing all suspected cases will involve additional resources, time, and money.

This research is the first study that predicts when HAPI is most likely to occur through the use of transactional data. The proposed approach integrates GS with RF and Braden Scale (GS-RF). This model considers real-time patient status changes from admission until HAPI occurrence by collecting the most relevant 60 risk factors, which include Braden Scale. GS-RF achieved the best AUC (91.20 ± 0.26) and G-mean (91.17 ± 0.26) compared to the state-of-the-art models that predict HAPI in the literature.

The use of a dynamic model allows for a symbiotic relationship between the care team and technology, which would not be possible if either were attempted alone. The care team provides real-time assessment of factors that cannot be adequately captured by technology without the input of data points; the care team in turn does not have the resources to sift through historical documents or evaluate changes over time. This collaborative model allows for further prioritization and escalation of preventive interventions when patients need it most but allows for de-escalation when appropriate. The level of prioritization can reduce the burden of care for the patient and potentially reduce disruptions in sleep; it can also reduce the tasks for the bedside nurse, which allows for appropriate level of care delivery. All at-risk patients will still receive prevention initiatives, but prioritizing patients who will benefit from additional interventions allows for the greatest value of care.

Future work will develop a multimodal ML model that combines HAPI images and patients’ diagnoses to predict HAPI time. Furthermore, the model’s performance can be enhanced by adopting metaheuristic algorithms to optimize the hyperparameters. Finally, this suggested approach will be integrated with another model to predict who will develop HAPIs before the occurrence. The data about the predicted patients with HAPI will be fed to the proposed model to predict HAPI time. Therefore, it will help revolutionize risk assessment by determining who is likely to develop HAPIs and when they are likely to develop.

## Figures and Tables

**Table 1:** Variables/risk factors used to predict days until HAPI occurrence (adapted from [8]).

Risk Factors	Labs	Demographics	Medical	Assessments	Medications	Diagnosis	Medical Devices
Additional Risk Factors	**Albumin**	Age	**Visiting** **Transitional Unit** **During** **Hospitalization**	**Blood Pressure** **Systolic**	**Opioids**	**Comorbidity**	Artificial Air Management
	**Blood Urea** **Nitrogen (BUN)**	Ethnic Group	**American Society of** **Anesthesiologists (ASA) Score**	**Blood Pressure** **Diastolic**	**Steroid Use**	**Depression**	**Face Mask**
	**C-reactive** **Protein**	**Race**	**Steroid History**	**BMI (Body Mass** **Index)**	**Stimuli** **Anesthesia**	**Diabetes**	**Nasal** **Cannula**
	**Creatine** **Serum**	**Sex**	**Number of** **Surgeries**	**Count of Glasgow Coma Score (GCS) Comment**	Stimuli Paralytics	Pressure Injury on Admission	Noninvasive Ventilation
	**Hemoglobin**		**Palliative** **Orders**	**Glasgow Coma Score**	Stimuli Sedation	Renal Failure	Pharyngeal
	**High MAP (Mean Arterial Pressure)**		**Number of** **Pressure** **Injuries**	Weight Loss	Stimuli Tracheostomy	Sepsis Diagnosis	**Room Air**
	**Lactate**		**Visiting ICU During** **Hospitalization (i.e., ICU During Encounter)**	**Patient Refusal** **to Change Position**	Vasopressor	Stroke History	Ventilator
	**Sodium**		**Prior Year** **Inpatient Visit Counter**	**Pulse Oximetry**			Feeding Tube
			**Length-of-stay at** **Emergency** **Department**	Skin Abnormality on Admission			
				**Body Temperature**			
Modifiable Risk Factors (Braden Scale)				**Overall Braden Scale, Braden Six Subscales (Sensory** **Perception, Skin Moisture,** **Activity Status,** **Mobility Status,** **Nutrition Status, and Friction, and Shear Status)**			

**Bold** risk factors: factors selected by recursive feature elimination.

**Table 2:** Number and percentage of records in each risk category.

Category	All Records	0–7 days (High Risk)	>7 days (Medium Risk)
**Number of** **Records (%)**	4619 (100%)	1698 (36.76%)	2921 (63.24%)

**Table 3:** Confusion matrix.

		Predicted HAPI Time	
		Medium Risk	High Risk
**Actual HAPI Time**	**Medium Risk**	TN	FP
	**High Risk**	FN	TP

**Table 4:** HAPI timing results of training with 10-fold cross-validation and testing.

Models			80% Training (10-Fold Cross-Validation)				20% Testing			
			Sensitivity	AUC	G-mean	FPR	Sensitivity	AUC	G-mean	FPR
Proposed Approach	GS-RF	Mean	89.08	91.23	91.20	6.62	89.33	91.20	91.17	6.94
		CL	0.16	0.09	0.10	0.10	0.49	0.26	0.26	0.31
Other Algorithms	RF	Mean	87.27	90.55	90.49	6.16	87.16	90.34	90.28	6.48
		CL	0.62	0.37	0.38	0.33	1.78	1.02	1.03	1.21
	MLP	Mean	85.48	89.09	89.02	7.30	86.12	89.26	89.18	7.61
		CL	0.31	0.14	0.14	0.16	0.82	0.35	0.36	0.46
	AdaBoost	Mean	48.22	66.44	63.88	15.35	48.70	66.71	64.20	15.28
		CL	0.38	0.18	0.24	0.17	0.74	0.35	0.46	0.40
	KNN	Mean	78.66	83.74	83.59	11.17	78.25	83.40	83.23	11.44
		CL	0.30	0.17	0.18	0.15	0.69	0.34	0.36	0.39
	DT	Mean	84.59	87.28	87.24	10.02	84.57	87.09	87.05	10.38
		CL	0.28	0.18	0.18	0.16	0.57	0.32	0.32	0.34
	LR	Mean	51.64	68.19	66.14	15.26	52.43	68.48	66.55	15.47
		CL	0.34	0.15	0.20	0.15	0.65	0.32	0.38	0.47
	SVM	Mean	46.75	68.88	65.21	8.99	47.53	69.20	65.69	9.12
		CL	0.45	0.19	0.28	0.19	0.65	0.34	0.44	0.38
	LDA	Mean	51.53	68.02	65.99	15.49	52.45	68.35	66.45	15.75
		CL	0.34	0.16	0.20	0.15	0.61	0.32	0.37	0.44

CL = confidence level at 95%.

**Table 5:** Statistical tests performed on the proposed method (GS-RF).

Performance	ANOVA		*t*-Test			
	All Methods (Figure 4)		GS-RF vs. RF (Figure 5)		GS-RF vs. MLP (Figure 6)	
	F-Value	*p*-Value	T-Statistic	*p*-Value	T-Statistic	*p*-Value
Sensitivity	3026.42	0.000	−6.07	0.000	6.50	0.000
AUC	4068.01	0.000	−4.36	0.000	8.74	0.000
G-mean	3860.58	0.000	−4.48	0.000	8.73	0.000
FPR	302.94	0.000	−1.96	0.053	−2.34	0.022

**Table 6:** Financial implications for a sample of 100 patients identified as HAPI when comparing the timing model (i.e., GS-RF) vs. current practice (i.e., providing prevention actions for all patients suspected as HAPI).

Prevention Actions Cost per Patient *	Patients Required for Prevention Actions per Day Based on the Detection Prevalence		Savings per Day (Number of Patients)	Savings per Year ($)
	Without the Timing Model, Detection Prevalence is 100%	With the Timing Model (GS-RF) Detection Prevalence (36.23 ± 0.36)		
$50 per patient per day	100	35–37	63–65	1,149,750–1,186,250
$75 per patient per day	100	35–37	63–65	1,724,625–1,779,375
$100 per patient per day	100	35–37	63–65	2,299,500–2,372,500

* Prevention cost per HAPI patient is between $50 and $100 in the United States in 2021 [1].

## Data Availability

These data were extracted from ChristianaCare Health systems’ databases in Delaware, United States and de-identified for the purpose of this research. The data are not available for the public as they are owned by ChristianaCare.
